# Let's Move It Together: A Review of Group Benefits in Joint Object Control

**DOI:** 10.3389/fpsyg.2018.00918

**Published:** 2018-06-07

**Authors:** Basil Wahn, April Karlinsky, Laura Schmitz, Peter König

**Affiliations:** ^1^Institute of Cognitive Science, Universität Osnabrück, Osnabrück, Germany; ^2^Department of Psychology, University of British Columbia, Vancouver, BC, Canada; ^3^School of Kinesiology, University of British Columbia, Vancouver, BC, Canada; ^4^Department of Cognitive Science, Central European University, Budapest, Hungary; ^5^Fakultät für Philosophie, Wissenschaftstheorie und Religionswissenschaft, Ludwig-Maximilians-Universität, Munich, Germany; ^6^Institut für Neurophysiologie und Pathophysiologie, Universitätsklinikum Hamburg-Eppendorf, Hamburg, Germany

**Keywords:** social cognition, joint action, social interaction, motor coordination, coordination strategies

## Abstract

In daily life, humans frequently engage in object-directed joint actions, be it carrying a table together or jointly pulling a rope. When two or more individuals control an object together, they may distribute control by performing complementary actions, e.g., when two people hold a table at opposite ends. Alternatively, several individuals may execute control in a redundant manner by performing the same actions, e.g., when jointly pulling a rope in the same direction. Previous research has investigated whether dyads can outperform individuals in tasks where control is either distributed or redundant. The aim of the present review is to integrate findings for these two types of joint control to determine common principles and explain differing results. In sum, we find that when control is distributed, individuals tend to outperform dyads or attain similar performance levels. For redundant control, conversely, dyads have been shown to outperform individuals. We suggest that these differences can be explained by the possibility to freely divide control: Having the option to exercise control redundantly allows co-actors to coordinate individual contributions in line with individual capabilities, enabling them to maximize the benefit of the available skills in the group. In contrast, this freedom to adopt and adapt customized coordination strategies is not available when the distribution of control is determined from the outset.

## 1. Introduction

Humans frequently coordinate their actions to jointly manipulate and control objects. These object-directed joint actions range from basic tasks such as carrying a table together (Sebanz et al., 2006) to complex ones such as flying an airplane (Hutchins, 1995). By controlling an object jointly, co-actors in a group may reach higher performance levels than individuals performing the same task alone: They may reach a *group benefit* (Reed et al., 2006; Wahn et al., 2016). However, controlling an object jointly also introduces additional coordination demands because co-actors need to predict or react to each other's actions and adjust their own action planning accordingly (Knoblich and Jordan, 2003; Sebanz et al., 2006; Vesper et al., 2017). Thus, joint object control introduces dependencies between co-actors because one actor's actions directly affect the actions of the other actor and vice versa.

**Figure 1:**
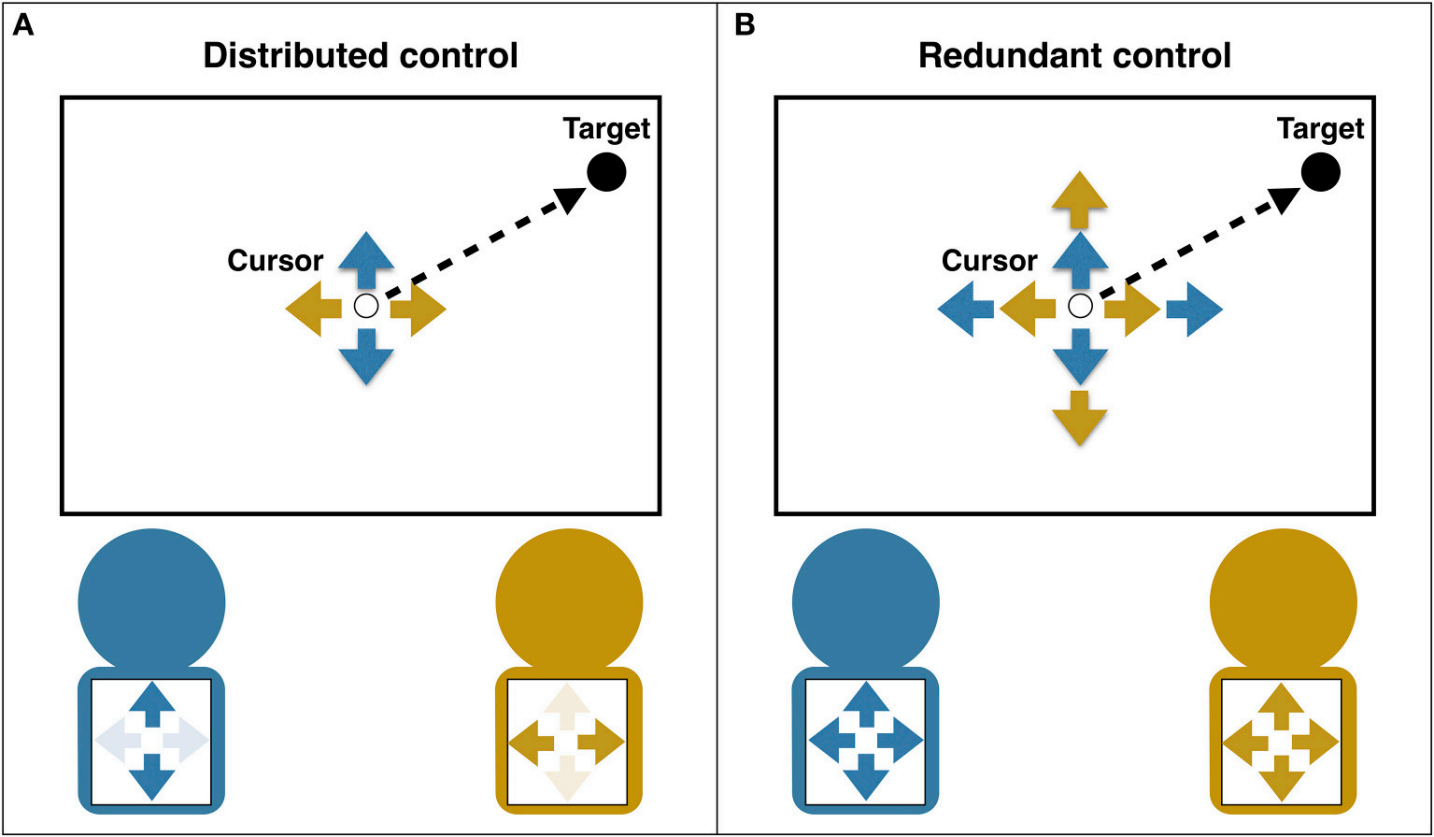
An example of a joint object control task in which two co-actors control the movements of a cursor with the goal to move it to a target location. **(A)** Distributed control: Control is divided between co-actors such that the left co-actor can move the cursor in the vertical dimension while the right co-actor can move the cursor within the horizontal dimension (see Wahn et al., 2016). **(B)** Redundant control: Both co-actors can move the cursor in the vertical and horizontal dimensions.

Research on object-directed joint action (henceforth referred to as “joint object control”) has investigated under which circumstances groups outperform individuals. In particular, researchers have identified different task types that determine whether the benefits of controlling an object together outweigh the costs of action coordination. In the present review, we discuss and compare two types of joint object control that have been shown to influence the emergence of group benefits: “distributed control” and “redundant control”. Distributed control refers to tasks where co-actors have predetermined complementary action possibilities. For instance, one co-actor controls object movement along the horizontal dimension while the other co-actor controls object movement along the vertical dimension. In contrast, redundant control refers to tasks where co-actors have the same action possibilities. For example, both co-actors can control object movement along the horizontal and vertical dimensions (see Figure 1). Note that in all of the studies that we consider in the present review, participants had visual access to the controlled object such that they could observe the combined effects of their own and their co-actor's actions on the object. In the following, we first review studies that have investigated distributed control. We then turn to studies that have investigated redundant control. Finally, we integrate the findings to determine factors that may explain differences in outcomes between the two task types and we point out directions for future research.

## 2. Distributed control

For distributed object control tasks, researchers have investigated how co-actors' access to information about each other's actions influences group benefits. In an early study, Knoblich and Jordan (2003) manipulated whether or not co-actors received information about each other's actions. Two co-actors were instructed to control a cursor on a computer screen in order to track an object that moved along the horizontal axis. Control over the cursor's movements was distributed such that one co-actor could press a key to increase the cursor's acceleration to the left whereas the other co-actor could press a key to increase acceleration to the right. Critically, co-actors either heard a tone whenever their co-actor pressed a key, or they did not receive any auditory information about each other's key presses. Joint performance was compared to an individual condition where individual participants controlled both movement directions bimanually. While individuals initially outperformed dyads, dyads eventually reached—but never exceeded—individual performance levels. Notably, joint performance improved only if co-actors received auditory information about each other's actions. Thus, individuals seem to have an initial performance advantage for this type of object control task.

Similar findings were observed when co-actors received haptic as opposed to auditory information about each other's actions (van der Wel et al., 2011). In the study by van der Wel et al. (2011), individuals and dyads moved a pole (similar to a pendulum) back and forth between two targets by pulling on two cords attached to the base of the pole. Control over the pole was either bimanual or distributed between two co-actors so that each co-actor controlled only one of the two movement directions by pulling one of the cords. Dyads reached a similar performance level as individuals, consistent with the study by Knoblich and Jordan (2003). The authors posited that receiving information about a co-actor's actions via the direct haptic coupling through the cords (in addition to seeing the pole move) was critical for dyads to achieve similar levels of performance as individuals (van der Wel et al., 2011). In a follow-up study, van der Wel et al. (2012) tested whether joint performance of the task would facilitate subsequent individual performance (and vice versa). However, they did not observe any transfer effects.

Taken together, these results suggest that when co-actors distribute control over an object to move it along one spatial dimension, co-actors need to receive information about each other's actions (beyond the visible outcomes of these actions) to attain performance levels akin to individuals performing the same task alone. Otherwise, individuals outperform dyads. Arguably, receiving such additional information allows co-actors to more easily simulate and predict each other's actions, thereby overcoming the problem of not being able to access each other's internal models (Wolpert et al., 2003; Sebanz et al., 2006).

Bosga and Meulenbroek (2007) investigated differences between individuals and dyads using a task where participants pressed force transducers to lift a virtual horizontal bar to a target area. In the individual condition, participants used two transducers to control both ends of the bar bimanually. In the joint distributed condition, each co-actor used a single hand and controlled only one end of the bar. In both conditions, participants could see the bar. Thus, co-actors could observe the combined effects of their actions on the controlled object but did not have direct information regarding the specific actions of their co-actor. In line with findings by Knoblich and Jordan (2003), Bosga and Meulenbroek (2007) found that individuals outperformed dyads: Individuals performed faster movement corrections while lifting the bar and were better at stabilizing the bar in the lifted position. These findings have been replicated in a follow-up study (Newman-Norlund et al., 2008).

Recently, Wahn et al. (2016) investigated distributed control across two spatial dimensions. Two participants controlled either the horizontal or vertical movement of a cursor via key presses. Their joint goal was to move the cursor from a start position to a target position as fast as possible. Reaching the target necessitated both coarse and fine types of control: Coarse control was needed for steering the cursor toward the target during the approach phase whereas fine control was needed for placing the cursor precisely on the target position during the homing-in phase. Compared to an individual bimanual condition, dyads did not attain a group benefit in the approach phase, but they did so in the homing-in phase. Thus, in contrast to the studies discussed above, dyads outperformed individuals even though co-actors were not provided with information about each other's actions (i.e., key presses) but could only observe the combined effects of their actions on the controlled object. As dyads exceeded individual performance levels only when a fine type of control was required, this suggests that group benefits for joint object control depend on the task demands (i.e., coarse vs. fine control).

In light of the reviewed findings, we suggest that the emergence of group benefits in distributed control tasks may be explained by the degree of coordination required. Specifically, if two co-actors distribute control over an object that moves within one spatial dimension such that they can steer the object in opposite directions, the actions of one co-actor immediately affect the actions of the other co-actor. This requires a high degree of interpersonal coordination. In contrast, when control is distributed across two spatial dimensions such as when one actor controls the horizontal and the other the vertical dimension, the actions of one co-actor do not directly constrain the actions of the other. This lowers coordination demands and facilitates group benefits.

Besides the degree of coordination required to control an object jointly, a further factor affecting group benefits are co-actors' interindividual skill differences. The similarity in co-actors' individual performance levels has been shown to predict group benefits in the two-dimensional object control task described above (Wahn et al., 2016): The more similar the co-actors' individual skills, the higher the group benefit when they perform together. There is also evidence that individuals do not benefit equally from interpersonal coordination (Mojtahedi et al., 2017). In particular, when two co-actors physically lifted and balanced an object by each grasping one of the two handles of the object, only the “worse” co-actor benefited (relative to her individual bimanual performance) whereas the “better” co-actor's performance tended to decrease when performing the task jointly (Mojtahedi et al., 2017). However, in line with Bosga and Meulenbroek (2007), the joint performance was still worse than the individual performance in this type of control task (Mojtahedi et al., 2017).

In sum, the majority of studies investigating joint tasks with distributed object control find that individuals outperform dyads (Knoblich and Jordan, 2003; Bosga and Meulenbroek, 2007; Newman-Norlund et al., 2008; Mojtahedi et al., 2017). Findings also indicate that joint performance depends on (1) whether co-actors receive specific information about each other's actions (beyond seeing their combined effects on the controlled object) (Knoblich and Jordan, 2003; van der Wel et al., 2011); (2) the degree of coordination required (e.g., coordination in one or two spatial dimensions); (3) the type of control required (i.e., a coarse or fine type of control) (Wahn et al., 2016); and (4) co-actors' interindividual skill differences (Wahn et al., 2016; Mojtahedi et al., 2017).

## 3. Redundant control

Group benefits have also been investigated using redundant object control tasks where two co-actors have the same sets of action possibilities and are free to exercise control redundantly or to flexibly distribute control. That is, despite the option to use all of their action possibilities, one co-actor may choose to use only a subset of her possible actions while the other co-actor may choose to use the complementary set. This type of voluntary distribution of control was demonstrated in a study by Reed et al. (2006). Dyads were instructed to accelerate an object within one spatial dimension toward a target position and then to decelerate the object until it stopped on the target. Control was redundant such that both co-actors could accelerate and decelerate the object. The authors found that dyads collaborated by having each co-actor focus on either accelerating or decelerating the object. Thus, co-actors chose to distribute control even though redundant control was possible. This coordination strategy successfully enabled dyads to reach a group benefit. These results suggest that in joint tasks where control is not distributed a priori, group benefits can be reached because co-actors can freely coordinate their preferred distribution of control. Of note, no such role specialization or group benefits were observed when participants performed the same task with a playback of human behavior (despite participants believing they acted with another person), suggesting that real, online interaction is necessary to reach a group benefit (Reed and Peshkin, 2008).

Further evidence that dyads adopt customized control strategies under redundant control conditions has been provided by Masumoto and Inui (2013, 2015). In a periodic force reproduction task, co-actors were required to jointly reproduce a target force (by continuously pressing force transducers) which varied periodically over time. While performing this task, they could see a visualization of the target force as well as their reproduced force on a computer screen. The authors found that dyads with redundant control achieved a more accurate performance than individuals performing the same task alone (Masumoto and Inui, 2013, 2015). Similar to the study by Reed et al. (2006), dyads used a distributed control strategy. That is, when one co-actor increased the exerted force, the other co-actor decreased her exerted force and vice versa. In a follow-up study, the authors manipulated co-actors' level of experience and found that whereas pairs with one experienced member initially showed greater levels of action coordination (i.e., more complementary force production) than pairs of two novices, the latter achieved similar performance levels after only one block of practice (Masumoto and Inui, 2014). Consistent with the benefits of practice in distributed control tasks discussed above (Knoblich and Jordan, 2003), these findings suggest that initial performance deficits (i.e., relative to individual performance or more skilled dyads) may be compensated for already within one experimental session.

In another set of studies, researchers investigated the effects of redundant object control on subsequent individual motor learning of a tracking task (Ganesh et al., 2014; Takagi et al., 2017). Dyads initially tracked the movements of a target object using a redundantly controlled cursor. Subsequently, they performed the same task individually. Individual performance on the task improved more after participants had practiced with a co-actor compared to when they had practiced alone, with a computer, or with a playback of a co-actor's performance (Ganesh et al., 2014). Thus, individuals benefited most from practicing with an interactive human partner. In a follow-up study, acting with a simulated interactive partner that was based on a human co-actor led to similar benefits in individual motor learning (Takagi et al., 2017). These findings, together with the results obtained by Reed and Peshkin (2008), suggest that using (simulated) interactive partners, rather than playback of human behavior, could be highly beneficial in real-world applications such as motor rehabilitation.

In sum, studies investigating redundant object control have shown that dyads outperform individuals, and that they distribute control when having redundant action possibilities (Reed et al., 2006; Masumoto and Inui, 2013, 2015). In addition, practicing a motor task jointly can benefit subsequent individual motor learning (Ganesh et al., 2014; Masumoto and Inui, 2017; Takagi et al., 2017).

## 4. Integration of findings and future directions

When comparing results for distributed and redundant control, findings suggest that dyads are more likely to attain a group benefit when they have redundant control. Why is redundant control more beneficial? We suggest that the opportunity to freely distribute control is a crucial factor as to whether or not group benefits are attained. Co-actors with redundant action possibilities have the option to distribute control in accordance with their coordination strategies and their individual capabilities, enabling them to combine their skills in the most efficient manner. Such customized control strategies are not available when the distribution of control is determined from the outset.

So far, a number of factors that have been investigated in distributed control have not yet been investigated in redundant control. In particular, it remains to be tested whether co-actors' performance in redundant control tasks is affected by the degree of coordination (e.g., coordination in one or two spatial dimensions), by the type of control required (i.e., a coarse or fine type of control) (Wahn et al., 2016), and by co-actors' interindividual skill differences (Wahn et al., 2016; Mojtahedi et al., 2017). Future research could also investigate how much time co-actors typically need to voluntarily distribute control, and whether the type of control distribution varies across dyads and across time.

An interesting factor that has not yet been investigated for either of the two types of control is group size. Does the size of a group benefit increase proportionally with the size of the group? Or is there an upper limit where the optimal group size has been reached such that further increasing the size will not lead to larger benefits? Another open question is how the social relationship between co-actors affects joint performance. Relatedly, a recent study on joint visual search found that the joint performance of two friends was better than that of two strangers (Brennan and Enns, 2015a). Furthermore, it would be worthwhile to investigate how individuals adjust their behavior to the specific co-actor with whom they are paired. It is likely that after first coordinating with one co-actor and then switching to a different one, individuals need to modify how they integrate the new co-actor's actions into their own action planning, possibly leading to initial decrements in joint performance.

A more technical direction for future research would be to introduce more informative measurements of joint performance. To date, the typical measure used to assess group benefits is the averaged performance difference between joint and individual conditions. Going beyond this measure, recent studies on joint visuospatial tasks have developed criteria to assess to what extent a group benefit can be ascribed to an actual collaboration between co-actors (Brennan and Enns, 2015b; Wahn et al., 2017, 2018a,b). That is, researchers have simulated a joint performance (based on the co-actors' individual performances) for which they assumed that co-actors act independently (i.e., do not collaborate). This simulated performance was then compared to the veridical joint performance. If veridical performance levels are higher than simulated performance levels, this suggests that co-actors did in fact collaborate. Similarly, future studies of joint object control could use measures that go beyond mere performance averages, thereby gaining valuable insight into how group benefits come about.

Finally, future research may explore whether the factors affecting group benefits in joint object control tasks are applicable to related real-world tasks. Areas of application range from aviation where pilot and co-pilot exercise joint control over an airplane, to motor rehabilitation where practice with another person might benefit subsequent individual motor learning (Takagi et al., 2017). In these contexts, research on joint object control may provide insights into how to circumvent individual motor limitations or how best to promote injury recovery.

## Author contributions

BW and AK literature research. BW, AK, and LS manuscript draft. BW, AK, LS, and PK manuscript revision.

### Conflict of interest statement

The authors declare that the research was conducted in the absence of any commercial or financial relationships that could be construed as a potential conflict of interest.
